# Human Papillomavirus (HPV)-Associated Squamous Cell Carcinoma In situ With Positive p16 and Ki-67 Immunohistochemical Stains in a Young Immunocompetent Patient

**DOI:** 10.7759/cureus.9673

**Published:** 2020-08-11

**Authors:** Aditi Chandra, Andrew Newman, Dustin Mullens, Christine C Lin

**Affiliations:** 1 Dermatology, Midwestern University, Arizona College of Osteopathic Medicine, Phoenix, USA; 2 Dermatology, Affiliated Dermatology, Scottsdale, USA; 3 Dermatology, Honor Health/Affiliated Dermatology, Scottsdale, USA

**Keywords:** squamous cell carcinoma of unknown primary, scc, human papillomavirus, hpv, hpv-associated sccis, immunocompetent, epidermodysplasia verruciformis, ev, p16, ki-67

## Abstract

There is an oncogenic role of human papillomavirus (HPV) in the development of premalignant and malignant skin cancers, especially squamous cell carcinomas (SCCs). Some of the major risk factors for SCC include older age, fair skin types, immunosuppression, ultraviolet radiation (UVR), history of epidermodysplasia verruciformis (EV), and co-carcinogenesis by the HPV. Our case report exemplifies a unique case of a low-risk, 34-year-old female who developed an HPV-associated squamous cell carcinoma in situ (SCCIS) on her left palmar hand, despite having none of the contributing risk factors. The biopsy also showed full-thickness keratinocyte atypia and increased mitotic activity throughout all the layers of the epidermis. Immunohistochemical stains showed strong and diffuse nuclear staining of p16 and Ki-67 throughout the SCCIS, confirming HPV etiology. We speculate that tumor development in our patient relied on the combined effects of UVR exposure, localized immunosuppression, and the co-carcinogenic effects of HPV infection.

## Introduction

Non-melanoma skin cancers (NMSCs) are the most common types of cancer in the United States, surpassing lung, breast, prostate, and colon cancers combined [1]. Additionally, their rates are increasing on an average of 4%-8% yearly, placing a significant burden on the health care system. NMSCs are composed of basal cell carcinomas (BCCs) and squamous cell carcinomas (SCCs). Some of the major risk factors for SCC include older age, immunosuppression, ultraviolet radiation (UVR), and fair skin types (Fitzpatrick skin types I-III). In addition to these risk factors, the clinical behavior and epidemiology of SCCs suggest a viral etiology [2].

Currently, the human papillomavirus (HPV) has been the focus of the etiological agent in SCC. HPV is a non-enveloped DNA virus that infects and depends on keratinocytes to replicate [3]. It is presumed that HPV gains entry via microabrasions on the skin or mucosal barriers, where it uses interactions between viral capsid proteins, host cell-surface glycosaminoglycans, and proteoglycans to adhere to and enter cells. It is not common for HPV DNA to integrate into the host DNA; however, some high-risk HPV types have been shown to integrate into the host genome [4]. There are over 100 types of HPV, the majority of which fall under the a, b, and g genera. a-HPV types typically infect mucosal epithelium, such as the cervix and oral cavity, while b and g-HPV types tend to infect cutaneous epithelium [5]. b-HPVs lead to tumor development by inhibition of apoptosis and interference of DNA repair mechanisms by viral E6 proteins. They also utilize viral E7 proteins to deregulate the host cell cycle and support invasive growth [6,7].

In the context of skin cancer, HPV infections can be divided into three categories: cutaneous, mucosal, and epidermodysplasia verruciformis (EV). EV is a rare cutaneous disorder that significantly increases the susceptibility of cutaneous HPV infections and subsequent skin malignancies [8]. The clinical manifestations of HPV infection depend on the viral genotype, immune status of the patient, and environmental carcinogens [9]. 

We present a unique case of a 34-year-old female with no prior history of Verruca vulgaris or known environmental exposures, such as working with children, immunosuppression, or EV, who presented with an HPV-associated squamous cell carcinoma in situ (SCCIS) on her left palmar hand. To the best of our knowledge, this is the only reported case of an HPV-associated cutaneous SCCIS in a patient this young and with none of the contributing risk factors.

## Case presentation

**Figure 1:**
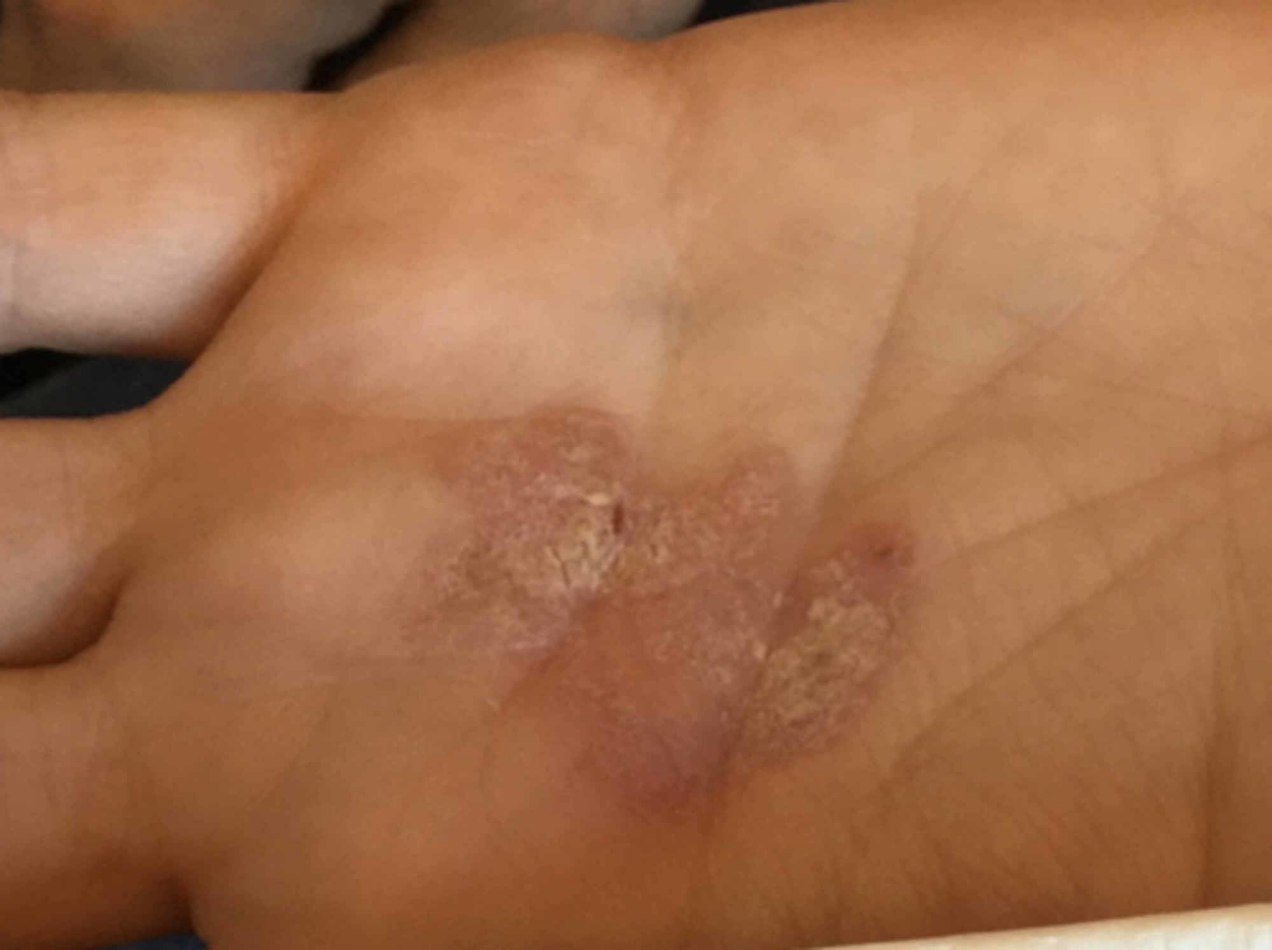
Left palmar hand with a 25-mm hyperkeratotic, erythematous, asymmetric plaque with well-defined, rolled borders and fissures.

We report a 34-year-old female with no comorbidities or previous history of malignancies, presenting with a moderately painful, progressive skin lesion on her left palmar hand that present for 5+ years. Physical examination revealed a 2.5-cm hyperkeratotic, erythematous, asymmetric plaque with well-defined, rolled borders and fissures (Figure 1). 

**Figure 2:**
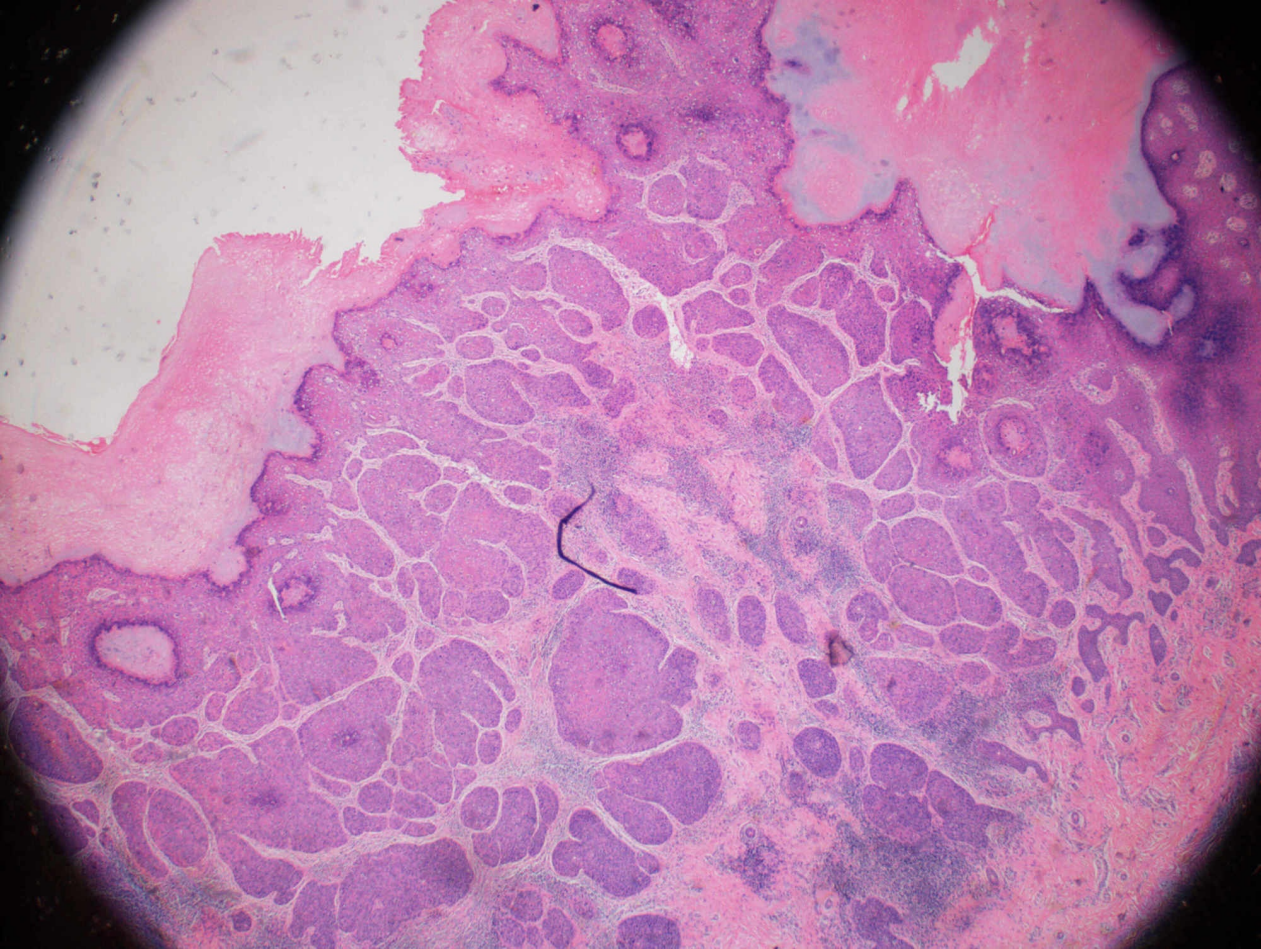
H&E-stained image of primary lesion biopsy. 2X objective view of tumor cells showing full-thickness keratinocyte atypia and mitotic activity throughout all the layers of the epidermis.

Shave biopsy revealed SCCIS with viral features, extending to the deep and lateral margins. The biopsy also showed full-thickness keratinocyte atypia and mitotic activity throughout all the layers of the epidermis (Figure 2). 

**Figure 3:**
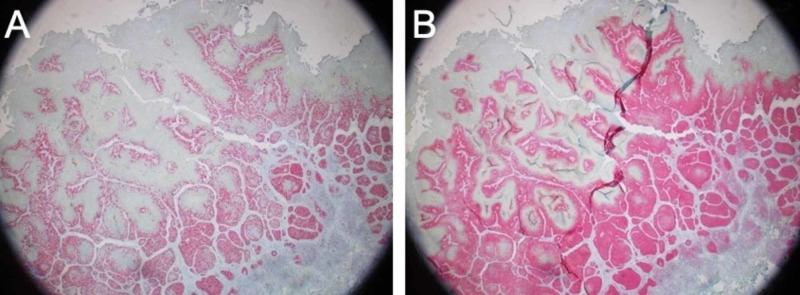
Immunohistochemical stains of primary lesion biopsy. 2X objective view exhibiting strong and diffuse nuclear staining of Ki-67 (A) and p16 (B) throughout the squamous cell in situ.

PAS stain was negative for fungal organisms. Immunohistochemical (IHC) stains showed strong and diffuse nuclear staining of Ki-67 and p16 throughout the squamous cell in situ, confirming HPV etiology (Figure 3). 

Mohs micrographic surgery was performed utilizing frozen tissue sections and H&E stains. The first two stages revealed residual carcinoma. The residual carcinoma was excised and submitted for processing, revealing no carcinoma on the third stage. Due to the large size of the defect, primary, complex, or adjacent tissue transfer closure was unachievable, and the wound was left to heal via secondary closure for six weeks. Subsequently, a skin graft was taken from the patient’s left medial arm and placed on the recipient site. The donor site was reconstructed via complex closure, and the recipient site defect measured at 38 mm x 85 mm. 

## Discussion

There is increasing evidence supporting the oncogenic role of HPV in the development of premalignant and malignant skin cancers [2,8,10]. Although no causative mechanism for the development of cutaneous SCCs (cSCCs) via an HPV infection is known, seropositive antibodies and viral DNA extracted from tumor tissue suggest a strong association. Additionally, some studies have found HPV to be more common in premalignant lesions, such as actinic keratosis (AK), than in SCCs, BCCs, benign lesions, or normal skin. This suggests that HPV may play a larger role in oncogenesis rather than tumor maintenance [11,12]. In a-HPV-associated carcinomas, viral oncoprotein E7 is expressed by the virus and inactivates retinoblastoma protein (pRb), which leads to a downstream upregulation of the tumor suppressor protein p16. Due to this well-established mechanism, p16 is a common IHC marker for the a-HPV infections in SCCs of the anogenital and mucosal regions. The common staining pattern for p16 is classified as positive when showing “block-type” diffuse nuclear and cytoplasmic staining. It is important to note that even though p16 is a commonly used marker for mucosal a-HPV-associated SCCs, further investigation needs to be done to establish a mechanistic relationship between p16 expression patterns in cutaneous b-HPV-associated SCCs. Nonetheless, the P16 marker has been shown in previous studies to strongly stain HPV-associated SCCs [13]. Another useful marker in estimating cell proliferation is Ki-67. Ki-67 is a protein expressed in the nuclei at all stages of the cell cycle, except for the G_0_ period, and thus an indication of the progression through the cell cycle of proliferating tumor cells. Additionally, HPV-associated SCCs are histologically characterized by full-thickness dysplasia of the epidermis, sometimes with the involvement of follicular epithelium.

In our case report, the patient’s histological results were consistent with an SCCIS, and the p16 staining in the IHC confirmed an HPV component. However, despite the strong histopathological picture of an HPV-associated SCCIS, the patient exhibited no contributing risk factors, which include a history of EV, immunosuppression, or older age.

EV is an extremely rare inherited skin condition that makes a person highly susceptible to infections caused by HPV. Symptoms can arise anywhere from infancy to puberty and consist of verrucae-like lesions distributed over sun-exposed areas of the body, such as hands, feet, face, and ears [8]. After the age of 30 years, about 30%-60% of these lesions can become malignant, commonly manifesting as SCCs [9]. Although the patient falls within this age group, she had no prior history or family history of EV. Additionally, studies comparing premalignant and malignant cutaneous lesions have exhibited a higher prevalence of EV-HPV-DNA extracted from actinic keratoses than from SCCs, suggesting a stronger involvement of EV-HPVs in the early stages of cutaneous oncogenesis specifically [8].

Another group of patients who are prone to SCCs is immunosuppressed patients. The HPV virus has been shown to elicit an antibody and cell-mediated immune response, with the T-cell response, specifically, playing a major role against HPV immunity [14]. Studies have demonstrated that patients with genetic defects in the quality and quantity of their T-cells, like Dock8 deficiency, have been shown to have a susceptibility to viral infections and the development of extensive cutaneous verrucae and SCCs [15]. Furthermore, patients with an acquired or iatrogenic immune deficiency, such as HIV-positive patients or organ transplant recipients, have been shown to have inefficient immunity against HPV and have a high incidence of cutaneous SCCs [16,17]. It is still unclear whether this predisposition to SCCs is due to their dampened immune system or if it is an underlying consequence of their immune defect. However, due to the increased risk in these populations, it is important to note the tendency of immunosuppressed patients developing HPV-associated cutaneous SCCs.

One risk factor that may have played a role in the patient’s development of cSCC is exposure to UVR. The relationship between UVR and cSCC development is not fully understood yet. Studies have shown patients with a history of painful sunburns had an increased risk of developing HPV-associated cSCCs [18]. The current hypothesis for this trend is that UV light creates localized immunosuppression by subduing local cell-mediated responses and thus promoting favorable conditions for HPV infection. This increased risk of HPV susceptibility was also seen in psoriasis patients treated with psoralen-UVA photochemotherapy when compared to their non-irradiated counterparts [19]. This strengthened the argument that UV exposure may aid in the development of HPV infections. Due to this influence of UVR exposure on skin cancer development, it is common to see the risk of cSCCs increase with age. The median age for SCCIS development is 73 years for immunocompetent patients and 63.5 years for immunocompromised patients, as reported by a large population-based study of non-genital SCCIS cases in a European population [20].

Studying HPV as a potential trigger in SCC carcinogenesis is important because it introduces the possibility for novel therapeutic and preventative measures for HPV infected patients. Currently, there are HPV vaccines that protect against cervical cancer, but they only cover nine of the many HPV serotypes. Additionally, current treatment options also do not eradicate active HPV infections. Future therapies should expand to include preventative vaccines for other HPV-induced cancers, including skin cancer, and enable virus eradication. This could lead to complete clearance of lesions and prevention of reoccurrences. 

## Conclusions

Our case report exemplifies a unique case of a low-risk patient who developed an HPV-associated SCCIS on her left palmar hand, despite having none of the contributing risk factors. At 34 years of age, she was well below the median age of developing SCCIS, and she presented with no history of immunosuppression or EV. Considering these findings, we speculate that tumor development in our patient relied on the combined effects of UVR exposure, localized immunosuppression, and the co-carcinogenic effects of HPV infection. 
